# Hair growth potential of *Salvia plebeia* extract and its associated mechanisms

**DOI:** 10.1080/13880209.2020.1759654

**Published:** 2020-05-18

**Authors:** Guang-Ri Jin, Yi-Lin Zhang, Jonathan Yap, William A. Boisvert, Bog-Hieu Lee

**Affiliations:** a Department of Food and Nutrition, College of Biotechnology and Natural Resources, Chung-Ang University, Anseong, Gyeonggi, Korea; b Center for Cardiovascular Research, John A Burns School of Medicine, University of Hawaii, Honolulu, HI, USA

**Keywords:** Human dermal papilla cells, anti-apoptotic, β-catenin, GSK3β, Bcl-2, Bax, ERK, Akt, C57BL/6 mice, hair regrowth

## Abstract

**Context:**

Although *Salvia plebeia* (SP) R. Brown (Labiatae) is known to possess various biological activities, the effects of SP on hair growth have not been elucidated.

**Objective:**

To investigate the hair growth potential of SP extract by using human dermal papilla cells (hDPCs) and C57BL/6 mice.

**Materials and methods:**

The entire SP plant sample was ground into powder and extracted with 99.9% methyl alcohol. Various concentrations of SP extract were added to hDPCs to evaluate the proliferation, migration, and factors related to hair growth and cycling. Effect of topical SP administration on hair regrowth was tested *in vivo* in male C57BL/6 mice for 21 days.

**Results:**

SP extract significantly increased the proliferation of cultured hDPCs at doses of 15.6 and 31.3 μg/mL compared to control group by 123% and 132%, respectively. Expression of hepatocyte growth factor increased while the level of TGF-β1 and SMAD2/3 decreased when treated with SP extract. At the molecular level, the extract activated Wnt/β-catenin signalling by raising β-catenin and phospho-GSK3β expression. SP extract also exerted anti-apoptotic and proliferative effects in hDPCs by increasing the Bcl-2/Bax ratio and activating cell proliferation-related proteins, ERK and Akt. Finally, the extract caused an induction of the anagen phase leading to significantly enhanced hair growth in treated male mice.

**Discussion and conclusion:**

Our results indicate that SP extract has the capacity to activate hDPCs into a proliferative state to promote hair growth. Further research is necessary to determine the bioactive components and their mechanisms of action responsible for SP-related hair growth effect.

## Introduction

The hair follicle, distributed over the human body, is one of the most complex mini-organs waiting to be explored. The mature hair follicle is composed of different concentric cylinders of epithelial cells, inner outer root sheaths, which are surrounding the hair shaft (Enshell-Seijffers et al. 2010). Mature hair follicles also contain dermal papilla which are derived from the mesenchymal cells. Each hair follicle consists of epithelial and mesenchymal parts and the interactions between them are essential for postnatal hair growth (Yang and Cotsarelis 2010). Dermal papilla cells (DPC), a group of dermal cells encapsulated by the epithelial matrix cells, are believed to generate signal factors that mediate the behaviour of keratinocytes in the follicle, thereby playing an essential function in hair cycling and growth (Kishimoto et al. 2000; Botchkarev and Kishimoto 2003). During the anagen phase, growth factors released from the DPC such as hepatocyte growth factor (HGF), vascular endothelial growth factor (VEGF), and insulin-like growth factor-1 (IGF-1) are believed to modulate the proliferation and differentiation of epithelial cells to form the hair shaft (Taylor et al. 2000; Oshima et al. 2001; Botchkarev and Kishimoto 2003; Enshell-Seijffers et al. 2010). Additionally, many studies have shown that transforming growth factor-β1/2 (TGF-β1/2) secreted by DPC results in apoptotic cell death of epithelial cells as well as anagen-to-catagen transition of hair follicle cycle (Inui et al. 2002; Soma et al. 2002).

According to clinical investigations, most hair growth disorders result primarily from changes in the three phases of hair follicle cycling, growth (anagen), regression (catagen), and rest (telogen) (Krause and Foitzik 2006). Androgenetic alopecia, a hair loss condition mediated by dihydrotestosterone, is one of the most common types of male-pattern hair loss, not only affecting 80% of men but also up to 50% of women. The key features of androgenetic alopecia are shortening of anagen and prolongation of telogen phases, accompanied by follicular miniaturization (Piraccini and Alessandrini 2014). Previous studies have suggested that the autocrine and paracrine factors from DPC and the increase of β-catenin in DPC, which is regulated by Wnt signal, are important for the maintenance of anagen phase and regeneration of hair follicle cycle (Kishimoto et al. 2000; Shimizu and Morgan 2004; Kwack et al. 2008). Minoxidil, the only drug approved by the US Food and Drug Administration for hair growth, is known to promote the survival of human dermal papilla cells (hDPCs) by activating extracellular signal-regulated kinase (ERK) and protein kinase B (also known as Akt) signalling, and prevent hDPCs apoptosis by increasing the ratio of Bcl-2/Bax (Han et al. 2004). Given the transient efficacy and widely reported side effects of minoxidil, the development of novel agents that promote hair growth safely and effectively will be beneficial to those affected by hair loss.

*Salvia plebeia* (SP) R. Brown (Labiatae) is an annual or biennial plant that grows widely throughout Asia (China, Korea, India, and Iran). In Korea, it has been used as an herbal traditional medicine for hepatitis, cough, menorrhagia, diarrhoea and haemorrhoids (Jung et al. 2009). SP contains flavonoids such as hispidulin, eupatorin, luteolin, nepetin, and 2′-hydroxy-5′-methoxybiochanin A, as well as phenolic compounds such as rosmarinic acid (Ai-Li and Chang-Hai 2006; Nugroho et al. 2012). Several reports and clinical studies have demonstrated that SP possesses various biological activities such as antimicrobial, antiallergic and hepatoprotective activities (Jin et al. 2008). However, there have been no reports on the effects of SP on DPC function or hair growth.

This study investigates the effect of SP extract on hair growth as well as its mechanism of action by using *in vivo* and *in vitro* models. For the mechanistic studies, cytokine and signalling pathways related to hair growth and cell cycling were investigated using cultured hDPCs. Furthermore, the effectiveness of SP extract on hair growth was tested *in vivo* using C57BL/6 mice as a model.

## Materials and methods

### Materials

Dulbecco’s modification of Eagle’s medium (DMEM) was obtained from Corning (Corning, NY, USA) and foetal bovine serum (FBS) was from Capricorn Scientific (Ebsdorfergrund, Germany). Penicillin and trypsin were purchased from Welgene (Daegu, Korea). Minoxidil, Triton X-100, Cell Counting Kit-8 (CCK-8), dimethyl sulfoxide (DMSO), haematoxylin and eosin (H&E) stain were from Sigma-Aldrich (St. Louis, MO, USA). Polyclonal antibodies for Bcl-2, Bax, phospho-Akt, Akt, phospho-ERK, ERK, β-catenin, phospho-β-catenin, Smad2/3 were obtained from Cell Signalling Technology (Danvers, MA, USA). Polyclonal antibodies for phospho-GSK3β, GSK3β, β-actin were purchased from Santa Cruz (Santa Cruz, CA, USA). RNeasy Mini Kit was obtained from Qiagen (Dusseldorf, Germany). A RevertAid First Strand cDNA Synthesis Kit, Maxima SYBR Green/ROX qPCR Mater Mix 2X were purchased from Thermo Fisher Scientific (Waltham, MA, USA).

### Preparation of *Salvia plebeia* extract

The dried whole parts of *S. plebeia* (SP) were purchased from the Korea Plant Extract Bank at the Korea Research Institute of Bioscience and Biotechnology (Daejeon, Korea) in 2017. Briefly, the whole plant sample of SP was collected from Gapcheon, Chungcheongnam-do, Korea during July 2001. The dried SP plants were ground into powder and extracted with 99.9% methyl alcohol for 3 days, after which the extract was filtered through 0.2 μm filter system (Corning, NY, USA). The filtered extract was concentrated in a Rotary Evaporator N-1000 SWD (SUNIL EYELA, Gyeonggi-do, Korea) at 45 °C, and then lyophilized to dryness. The sample of SP extract was dissolved in dimethyl sulfoxide (DMSO) for the study and stored at 4 °C until use.

### Cell culture

Human dermal papilla cells (hDPCs) were purchased from CEFO BIO (Seoul, Korea) and were maintained in DMEM supplemented with 10% FBS and 1% penicillin–streptomycin. Cells were incubated at 37 °C in a humidified atmosphere of 5% CO_2_. Cells were cultured for five passages before experiment.

### Cytotoxicity assay

The cytotoxic effects of SP extract on proliferating hDPCs were determined by Cell Counting Kit-8 (CCK-8). Cells (5 × 103 cells/well) were seeded into 96-well culture plates and incubated at 37 °C for 24 h. Cells were cultured with 100 μL of FBS-free DMEM for 24 h and then various concentrations of SP extract (3.9, 7.8, 15.6, 31.3, 62.5, 125, 250 μg/mL) were added to each well. FBS-free DMEM, 10 μM minoxidil, and triton X-100 were used as negative controls (NC), positive controls (PC), and blanks, respectively. After 24 h of incubation, 10 μL CCK-8 solution was added to each well and incubated at 37 °C for 4 h. Absorbance was measured at 450 nm (test wavelength) and 650 nm (reference wavelength) by using microplate spectrophotometer (Multiskan™ GO; Thermo Scientific, Waltham, MA, USA). The results were represented as percentages of NC group.

### Cell migration assay

Migration assays were conducted as described previously (Liang et al. 2007). Briefly, hDPCs were cultured in 6-well plates and grown to 90% confluence in DMEM containing 10% FBS and 1% penicillin. 200 μL pipette tips were used to scratch the monolayer cells in a straight line and then washed by Dulbecco’s phosphate buffered saline (DPBS). Cells were treated with 7.8, 15.6, 31.3 μg/mL of SP extract, respectively. After incubation for 24 h, the widths of the scratch were photographed by optical microscope (Leica DM500, Wetzlar, Germany; 40× magnification).

### Real-time RT-PCR

Real-time RT-PCR was used to analyse the relative quantities of cDNA of transforming growth factor-beta1 (TGF-β1) and hepatocyte growth factor (HGF). hDPCs were cultured in 6-well plates and were treated with FBS-free DMEM (NC), 10 μM minoxidil (PC), and various concentrations of SP extract for 24 h, respectively. Total RNA was extracted using an RNeasy Mini Kit according to the manufacturer’s instructions and cDNA was synthesized from 1 μg total RNA using an RevertAid First Strand cDNA Synthesis Kit. The reaction was performed in Piko-real™ 96 real-time PCR system (Thermo Fisher Scientific Inc., Waltham, MA, USA) using Maxima SYBR Green/ROX qPCR Mater Mix. The following protocol for both TGF-β1 and HGF was used: pre-denaturation at 95 °C for 10 min, 40 cycles at 95 °C for 15 s, 60 °C for 30 s, 72 °C for 30 s. Primer pairs used in this study are shown in Table 1.

**Table 1.** Primer pairs used for quantitative real-time RT-PCR.

Target genes	Primer	Sequence
TGF-β1	Forward	5′-GCC CTG GAC ACC AAC TAT TG-3′
	Reverse	5′-GTC CAG GCT CCA AAT GTA GG-3′
HGF	Forward	5′-AGA AAT GCA GCC AGC ATC AT-3′
	Reverse	5′-CAC ATG GTC CTG ATC CAA TC-3′
GAPDH	Forward	5′-GGA AGG TGA AGG TCG GAG TC-3′
	Reverse	5′-CTC AGC CTT GAC GGT GCC ATG-3′

### Western blot analysis

hDPCs were incubated with various concentrations of SP extract for 24 h and then washed three times with DPBS. Proteins were extracted using ice-cold RIPA lysis buffer (Millipore, Billerica, MA, USA). Cell debris was removed by centrifugation and the resulting supernatants were collected and protein concentration was determined using BCA protein assay (Thermo Scientific, Bartlesville, OK, USA). The protein was separated on 10% SDS-polyacrylamide gel and then transferred to nitrocellulose membranes (Hybond, GE Healthcare Life Sciences, Little Chalfont, UK). Membranes were incubated with specific primary antibodies overnight at 4 °C. After washing, the membranes were reacted with diluted enzyme-linked secondary antibodies for 1 h at room temperature. Antibody-antigen complexes were visualized by using enhanced chemiluminescence (ECL) system (SuperSignal, Thermo Scientific, Rockford, IL, USA).

### Experimental animals

Male C57BL/6 mice (5-week-old) were purchased from Daehan Biolink Co., Ltd (Eumsung, Chungbuk, Korea) and then allowed to adapt for 1 week before the study, with food and water provided *ad libitum*. The mice were housed in cages at a temperature of 23 ± 3 °C, relative humidity of 45–60% and were exposed to a 12 h day and night photoperiodic cycle. The experimental protocol was reviewed and approved by the Institutional Animal Care and Use Committee of Chung-Ang University (approval number: 2017-00041).

### Experimental animal models treated with *Salvia plebeia* extract

Eighteen animals in 3 randomized groups (n = 6) were used for the study of hair promoting activity of SP extract. Hairs were shaved from the dorsal skin of all mice using an animal clipper and hair follicles were synchronized using wax stripping. From 1 day after hair removal, 100 μL of vehicle (DMSO), 3% minoxidil (Hyundai Pharm Co., Ltd. Seoul, Korea) or SP extract (1000 μg/mL concentration dissolved in DMSO) was topically applied daily on the dorsal skin of mice for 3 weeks. Visible hair growth was photographed at day 0, 7, 14 and 21, respectively.

### Hair length determination

Regrown hairs were plucked randomly from shaved dorsal centre parts of each mice on the 14th and 21st day. After plucking, the average hair length was then measured manually.

### Histological analysis

After 21 days, the dorsal skins of mice were surgically removed and fixed with 10% formalin. The fixed samples were processed by ascending series of graded ethanol and cleared in xylene. After that samples were embedded in paraffin blocks using standard techniques, followed by being cut longitudinally into sections. Sections (5 μm) were stained with haematoxylin and eosin (H&E) and observed through an optical microscope (100× magnification).

### Statistical analysis

The experimental data are expressed as mean ± standard deviation (SD). Experiments were performed in triplicate. The Student’s *t*-test or Duncan’s multiple range test for *post hoc* comparisons were used to analyse the data. Statistical analyses were performed in SPSS, version 23.0 (SPSS Inc., Chicago, IL, USA). Statistical significance was defined as *p < 0.05, **p < 0.01, and ***p < 0.001.

## Results

### *Salvia plebeia* extract promotes the proliferation of hDPCs

**Figure 1.**
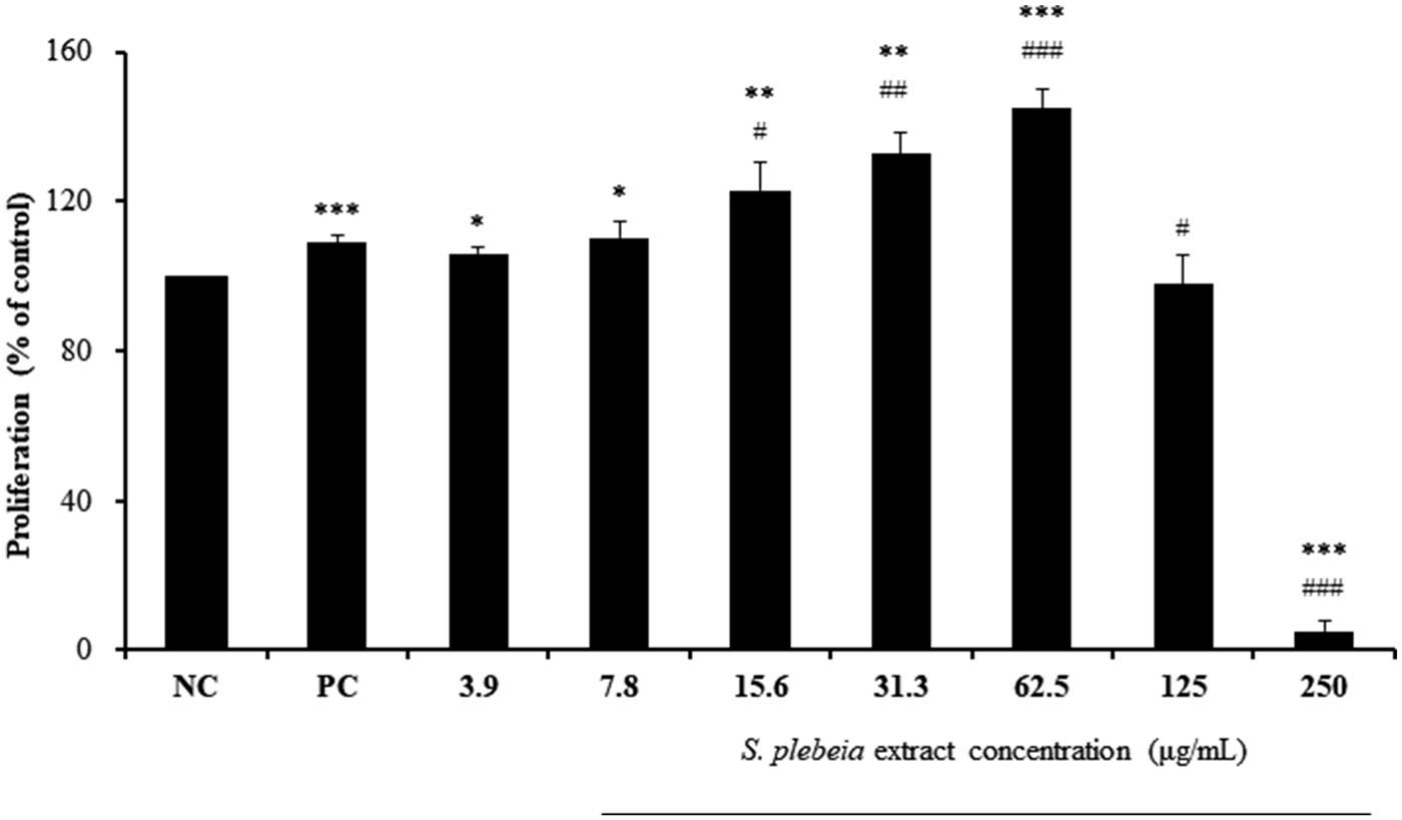
Effect of *S. plebeia* extract on the proliferation of hDPCs as determined by CCK-8 assay. The data shown are the mean ± SD (n = 3). NC: negative control of hDPCs treated with DMEM; PC: positive control of hDPCs treated with 10 μM minoxidil. *p < 0.05, **p < 0.01, ***p < 0.001 compared with NC; ^#^*p* < 0.05, ^##^*p* < 0.01, ^###^*p* < 0.001 compared with PC.

Cytotoxicity assays were performed to determine the effects of SP extract on the proliferation of hDPCs. SP extract significantly enhanced the proliferation of hDPCs in a dose-dependent manner at the concentrations of 3.9, 7.8, 15.6, 31.3 and 62.5 μg/mL compared with NC group. The treatment of both SP extract at the concentrations of 15.6 and 31.3 μg/mL showed stronger proliferation by 123.03 ± 7.67% and 132.78 ± 5.38%, respectively, than the PC group (109.07 ± 1.65%), while 62.5 μg/mL concentration exhibited the greatest proliferation by 144.99 ± 4.88% compared to both NC and PC groups (*p* < 0.001). At higher concentrations of 125 and 250 μg/mL, however, there was a decrease in proliferation compared with NC group (Figure 1), possibly suggesting cytotoxicity at these high concentrations. The results indicated that SP extract concentrations from 3.9 to 62.5 μg/mL were considered for the hDPCs, and were used in subsequent experiments.

### *Salvia plebeia* extract increases the migration of hDPCs

**Figure 2.**
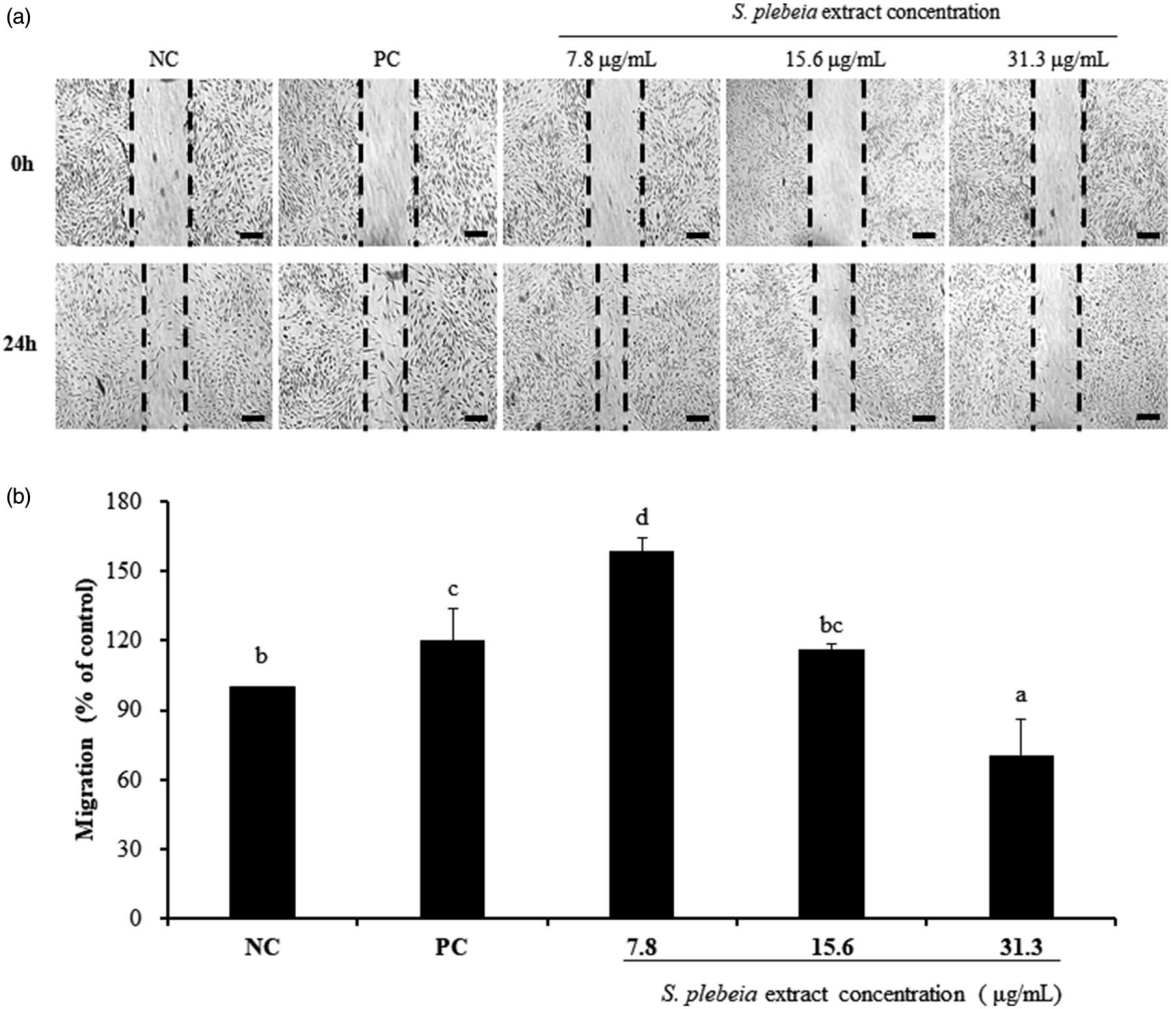
Effect of *S. plebeia* extract on the migration of hDPCs measured by scratch assay. (a) Migration stimulating effect of *S. plebeia* extract; (b) Migration percent of *S. plebeia* extract. Magnification bars are 100 μm. The data shown are mean ± SD (n = 3). Values with different superscripts are significantly different at *p* < 0.05 by Duncan’s multiple range test. NC: negative control of hDPCs treated with DMEM; PC: positive control of hDPCs treated with 10 μM minoxidil.

The effect of SP extract on hDPC migration was measured by scratch wound-healing assay (Figure 2(a,b)). Treatment with 7.8 μg/mL SP extract was found to markedly increase the migration of hDPCs by 158.45 ± 5.62% in comparison with both NC and PC groups (120.16 ± 13.53%; *p* < 0.05). SP extract at the concentration of 15.6 μg/mL also stimulated the migration rate of hDPCs (116.35 ± 2.01%) compared with NC, however these changes were not statistically significant. On the contrary, high concentrations of SP extract (31.3 μg/mL) hampered wound-healing responses of hDPCs. This result suggests that relatively low concentration of the SP extract, such as 7.8 and 15.6 μg/mL concentrations, enhances the process of wound closure compared with the untreated hDPCs.

### Induction of hair growth factors by *Salvia plebeia*

**Figure 3.**
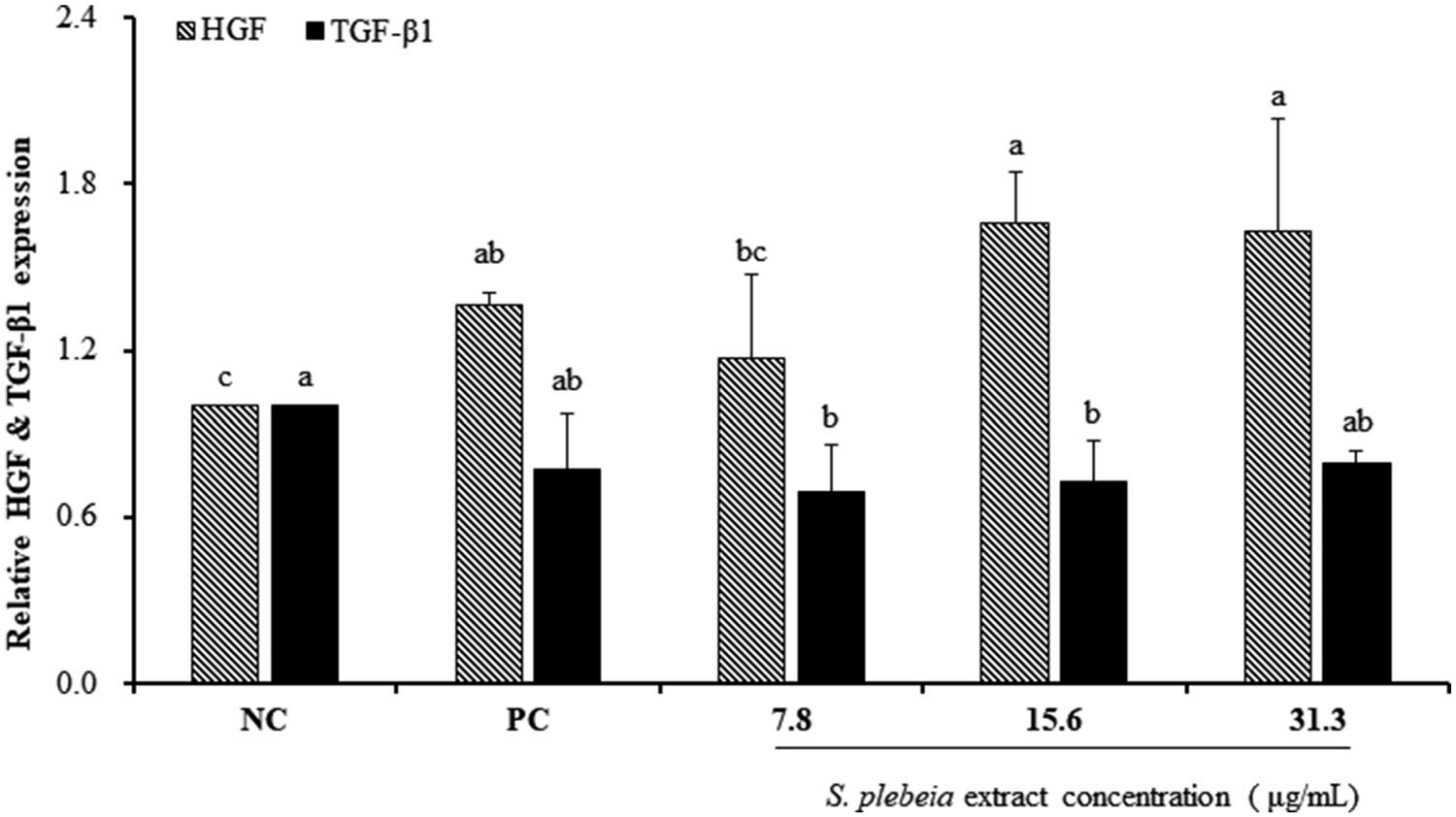
Effects of *S. plebeia* extract on the expression of HGF and TGF-β1 related to hair growth with the treatment of hDPCs. The data shown are mean ± SD (n = 3). Values with different superscripts are significantly different at *p* < 0.05 by Duncan’s multiple range test. NC: negative control of hDPCs treated with DMEM; PC: positive control of hDPCs treated with 10 μM minoxidil.

The relative expression levels of known hair growth mediators were measured by real-time RT-PCR in cultured hDPCs (Figure 3). Compared to the NC group, SP extract at concentrations of 15.6 and 31.3 μg/mL significantly upregulated the expression level of HGF by 1.66-fold and 1.63-fold, respectively (*p* < 0.05). In contrast, treatment with 7.8 and 15.6 μg/mL of SP extract significantly suppressed the expression levels of TGF-β1 in hDPCs as compared to the NC group (*p* < 0.05). These results suggest that the expression of HGF and TGF-β1 released from hDPCs might be regulated by SP extract treatment.

### *Salvia plebeia*-mediated regulation of signalling molecules in hDPCs

**Figure 4.**
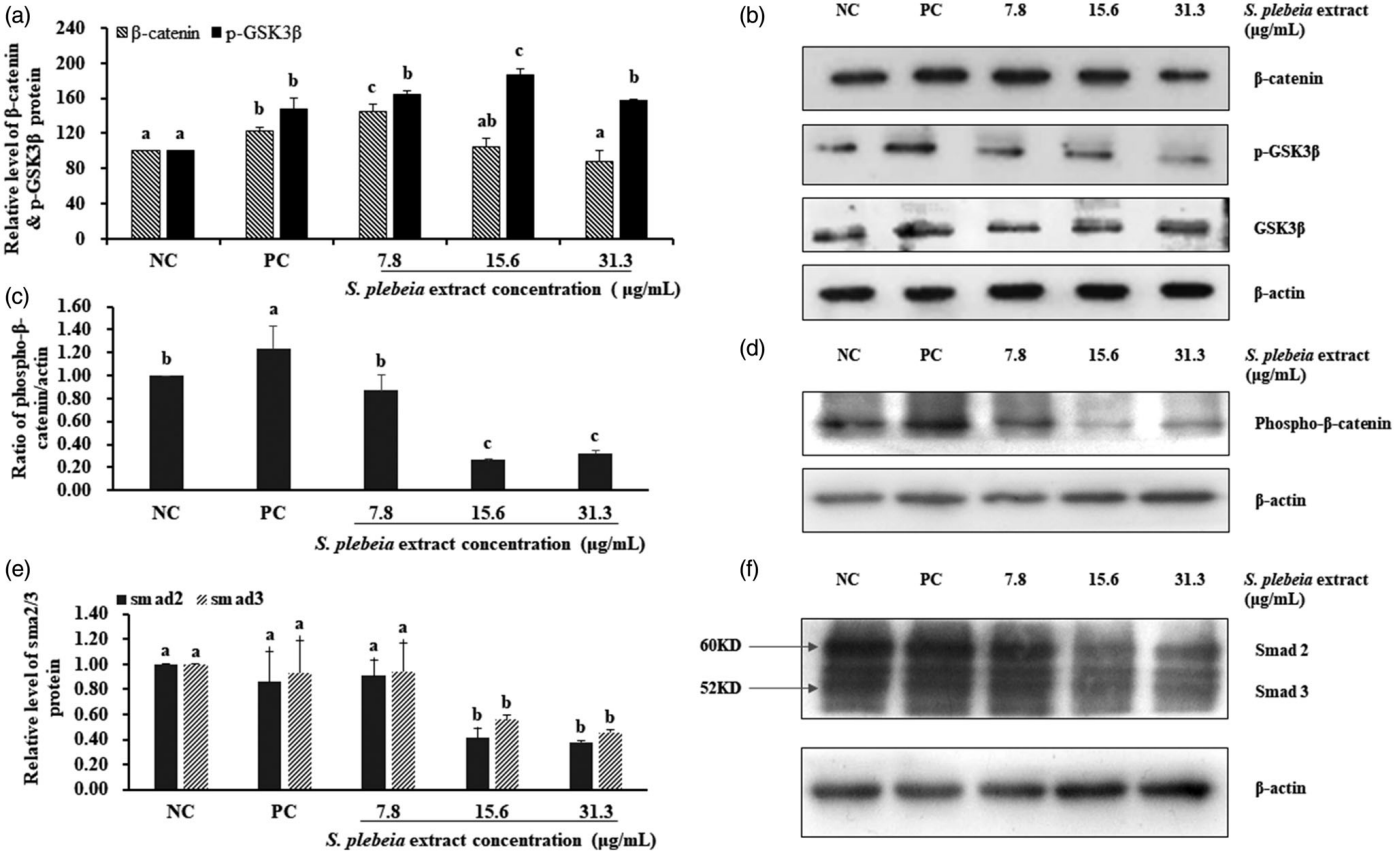
*Salvia plebeia* extract increased the phosphorylation of β-catenin and phospho-GSK3β in hDPCs analysed by western blot. (a) Graph of protein expression. (b) The protein level of β-catenin, phospho-GSK3 and β GSK3β. (c) Graph of protein expression. (d) The protein level of phospho-β-catenin. (e) Graph of protein expression. (f) The protein level of Smad2/3. The data shown are mean ± SD (n = 3). Values with different superscripts are significantly different at *p* < 0.05 by Duncan’s multiple range test. NC: negative control of hDPCs treated with DMEM; PC: positive control of hDPCs treated with 10 μM minoxidil.

To investigate the mechanisms related to the proliferative effects of SP extract in hDPCs, signalling molecules were analysed using western blot assays. Wnt signalling is known to play a key role in the proliferation and differentiation of hair follicle, as well as the regulation of hair growth cycle. Wnt proteins can activate the canonical Wnt signalling (Wnt/β-catenin signalling) pathway which contributes to the prevention of regression phase of hair cycle as well as the regeneration of hair follicle (Enshell-Seijffers et al. 2010; Teo and Kahn 2010). Treatment with 7.8 μg/mL SP extract significantly increased the total level of β-catenin compared with NC and PC groups by 144.50 ± 8.02% (*p* < 0.05). Expression of phospho-GSK3β was significantly increased in hDPCs treated with 15.6 μg/mL SP extract compared with NC group (*p* < 0.05), whereas the expression of total GSK3β was not elevated (Figure 4(a,b)). The protein expression of phospho-β-catenin in PC group compared with NC group increased significantly by 123% (*p* < 0.05). However, treatment with 7.8 μg/mL SP extract tended to decrease the total level of phospho-β-catenin compared with NC group by 87%, although this was not significant (Figure 4(c,d)).

We also determined whether SP extract can inhibit the activation of the Smad2/3 cascades. The results show that treatment with minoxidil resulted in a significant decrease in Smad2/3 activation by 86% and 93%, respectively, as expected. On the other hand, treatment with 15.6 μg/mL SP extract markedly decreased Smad2/3 expression by hDPCs by 41% and 56%, respectively, in comparison to both NC and PC groups (*p* < 0.05). Similar decrease in Smad2/3 levels was observed at 31.3 μg/mL SP extract concentration by 38% and 45%, respectively (Figure 4(e,f)).

**Figure 5.**
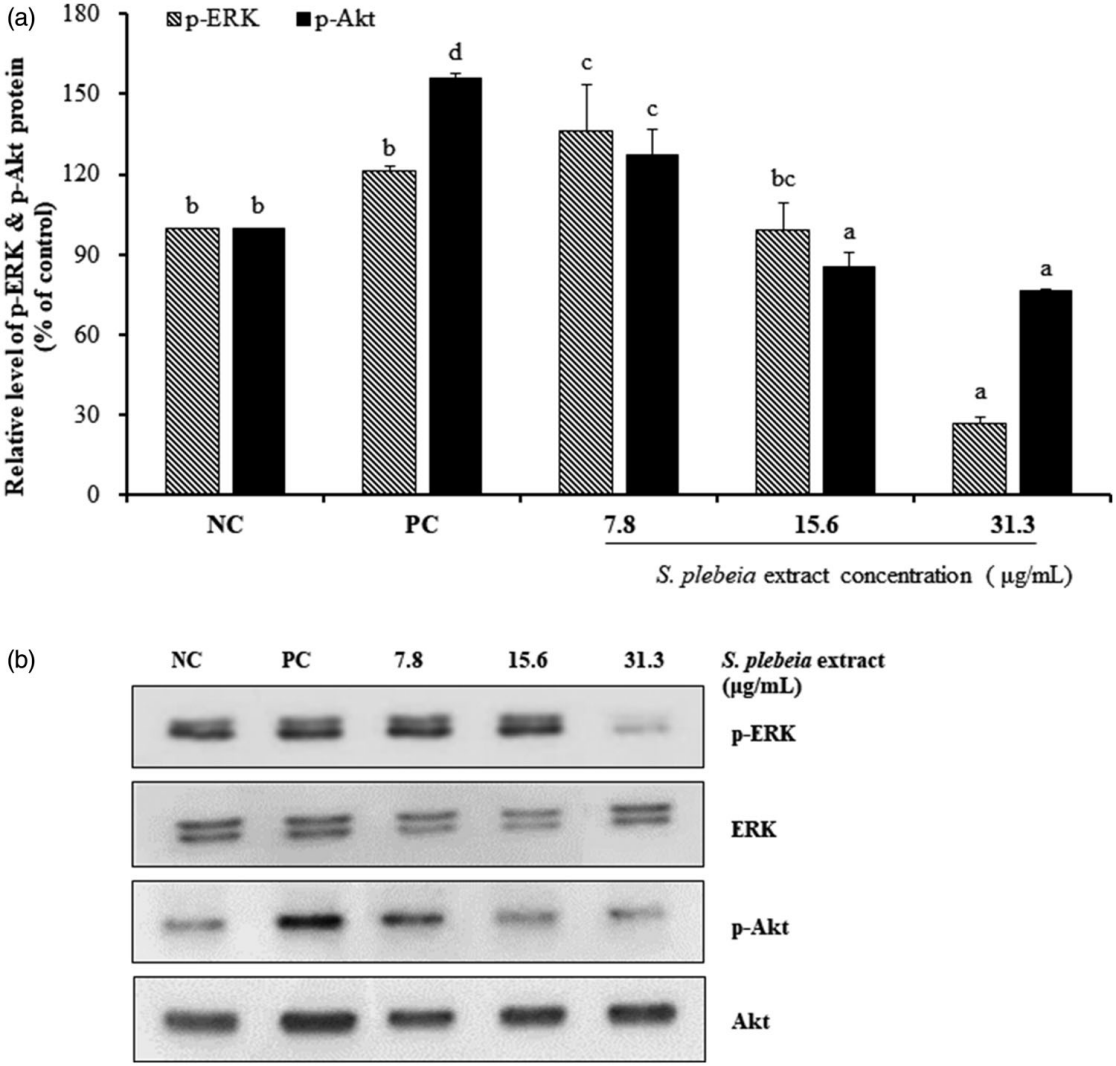
*Salvia plebeia* extract increased the phosphorylation of phospho-ERK and phospho-Akt in hDPCs analysed by western blot. (a) Graph of protein expression. (b) The protein level of ERK, phospho-ERK, Akt and phospho-Akt. The data shown are mean ± SD (n = 3). Values with different superscripts are significantly different at *p* < 0.05 by Duncan’s multiple range test. NC: negative control of hDPCs treated with DMEM; PC: positive control of hDPCs treated with 10 μM minoxidil.

**Figure 6.**
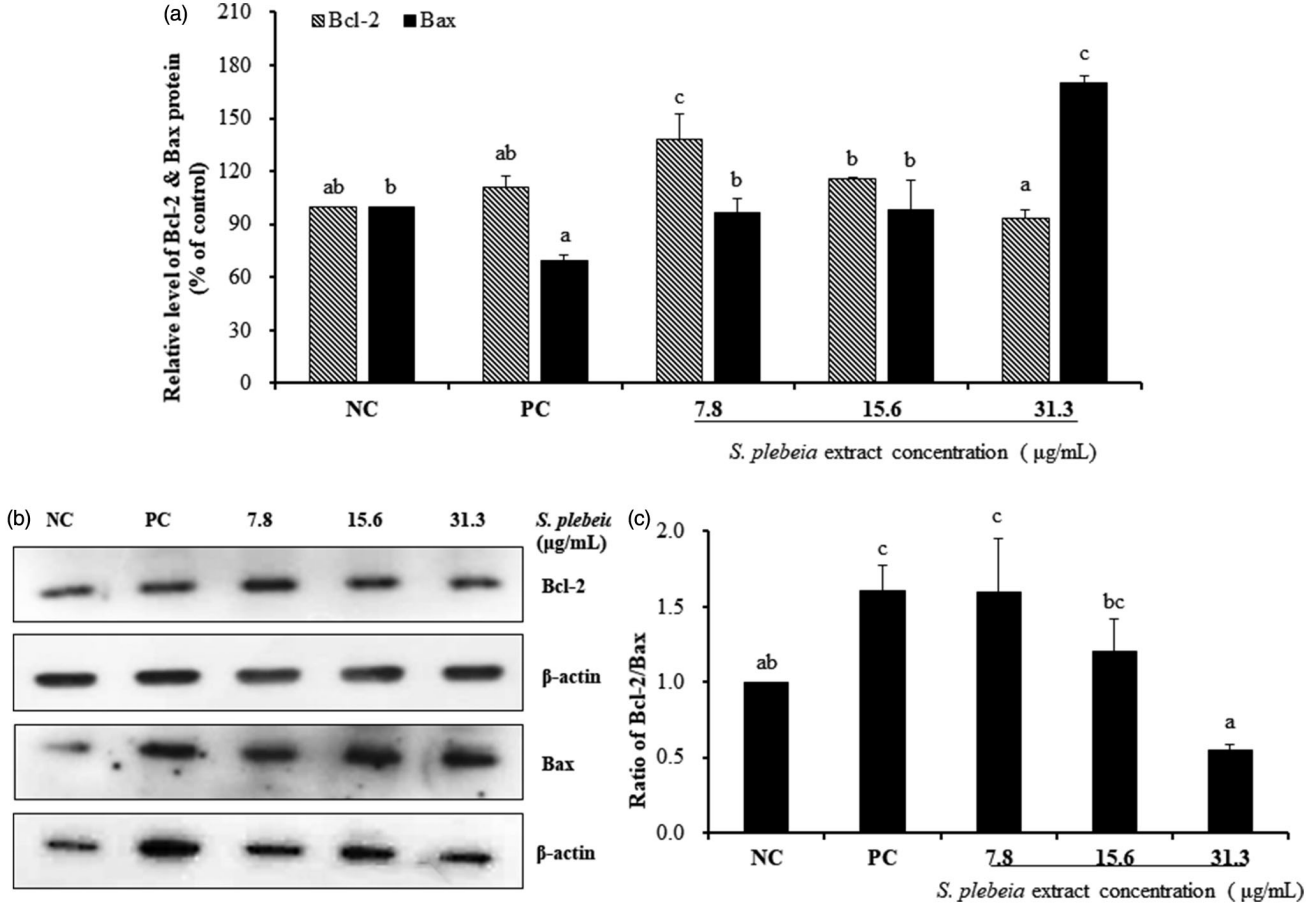
The effect of *S. plebeia* extract on the expression of Bcl-2 and Bax proteins in hDPCs analysed by western blot. (a) Graph of protein expression; (b) The protein level of Bcl-2 and Bax; (c) The ratio of Bcl-2/Bax. The data shown are mean ± SD (n = 3). Values with different superscripts are significantly different at *p* < 0.05 by Duncan’s multiple range test. NC: negative control of hDPCs treated with DMEM; PC: positive control of hDPCs treated with 10 μM minoxidil.

In comparison with NC and PC groups, SP extract at concentration of 7.8 μg/mL markedly enhanced the phospho-ERK expression by 136.05 ± 17.46% (*p* < 0.05). Levels of phospho-Akt also were increased significantly (by 127.31 ± 9.28%) compared with NC group after 24 h SP extract treatment at concentration of 7.8 μg/mL (*p* < 0.05), but were lower compared to the PC group (155.86 ± 2.06%; Figure 5(a,b)). The role of Bcl-2 family proteins in cell survival and apoptosis is well established. Bcl-2 protein expression increased significantly by 138.39 ± 14.18% with 7.8 μg/mL of SP extract compared with NC and PC groups (*p* < 0.05). On the other hand, Bax protein expression did not decrease after 24 h treatment of SP extract. This meant that the ratio of Bcl-2/Bax was still increased by 1.60-fold in 7.8 μg/mL SP extract group compared with NC group (*p* < 0.05; Figure 6(a–c)). Overall, these results suggest that the effect of SP extract on hair growth may be through the upregulated expression level of β-catenin as a result of suppressed GSK3β activity. Moreover, the activation of ERK and Akt pathways, as well as increased Bcl-2/Bax ratio, is likely to contribute to the proliferation of hDPCs when the cells are treated with lower doses of the SP extract.

### *Salvia plebeia* extract stimulates hair growth and hair follicle formation in C57BL/6 mice

**Figure 7.**
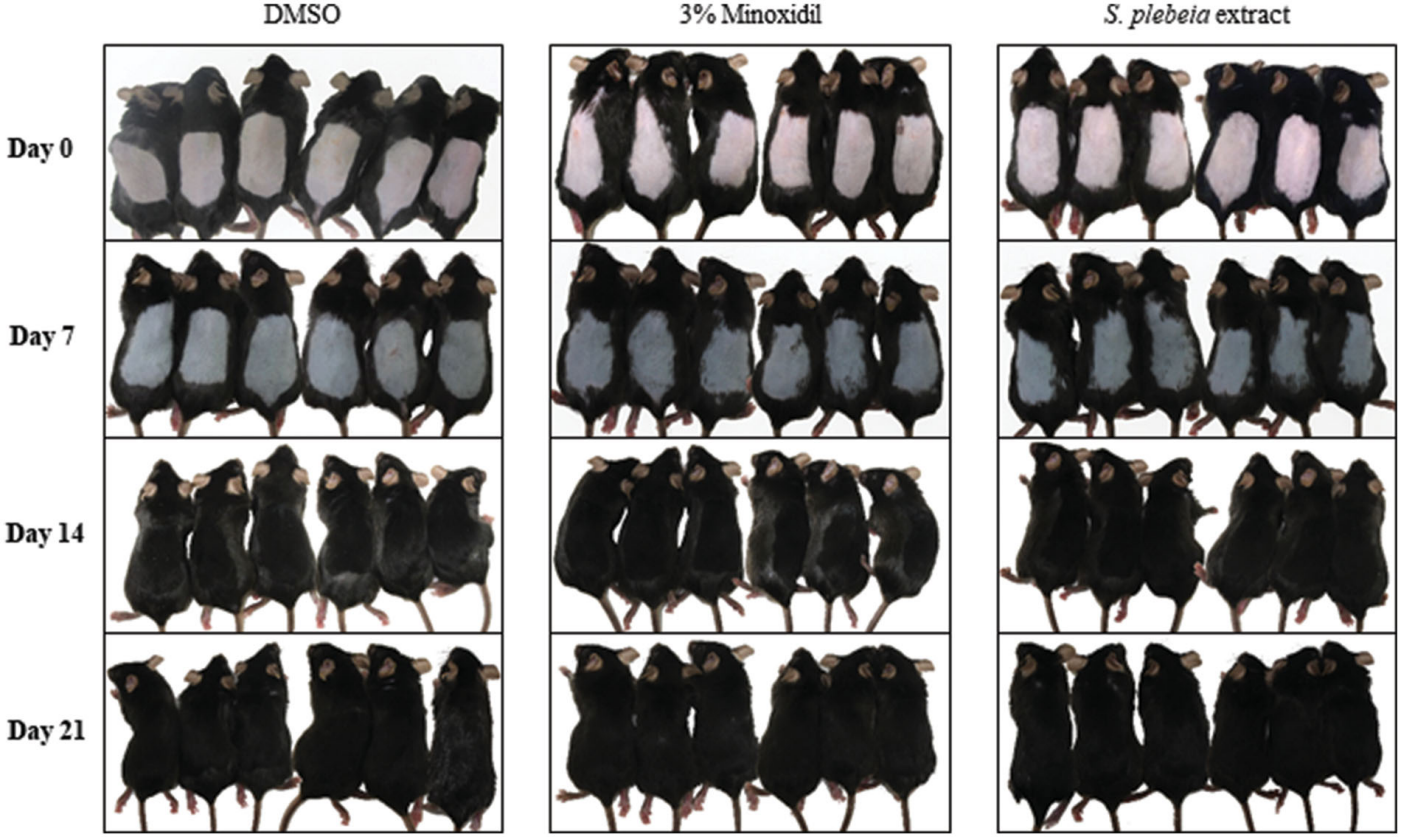
The effect of *S. plebeia* extract on anagen phase induction in C57BL/6 mice. After shaving, the back skin was treated with DMSO (vehicle), 3% minoxidil or *S. plebeia* extract (1000 μg/mL dissolved in DMSO) and photographed at 0, 7, 14 and 21 days. N = 6 per group.

SP extract was topically applied daily on the shaved dorsal skin of mice for 21 days and hair regeneration was observed. One week after treatment, all groups showed black pigmentation indicating transition of hair follicles from telogen to anagen phase. However, newly regrown hairs were observed only on the back skin of mice treated with minoxidil and SP extract groups. After 2 weeks, the dorsal skin hair in minoxidil and SP extract groups were completely recovered, whereas in the vehicle group bare patches could be still observed on the back of depilated areas (Figure 7).

**Figure 8.**
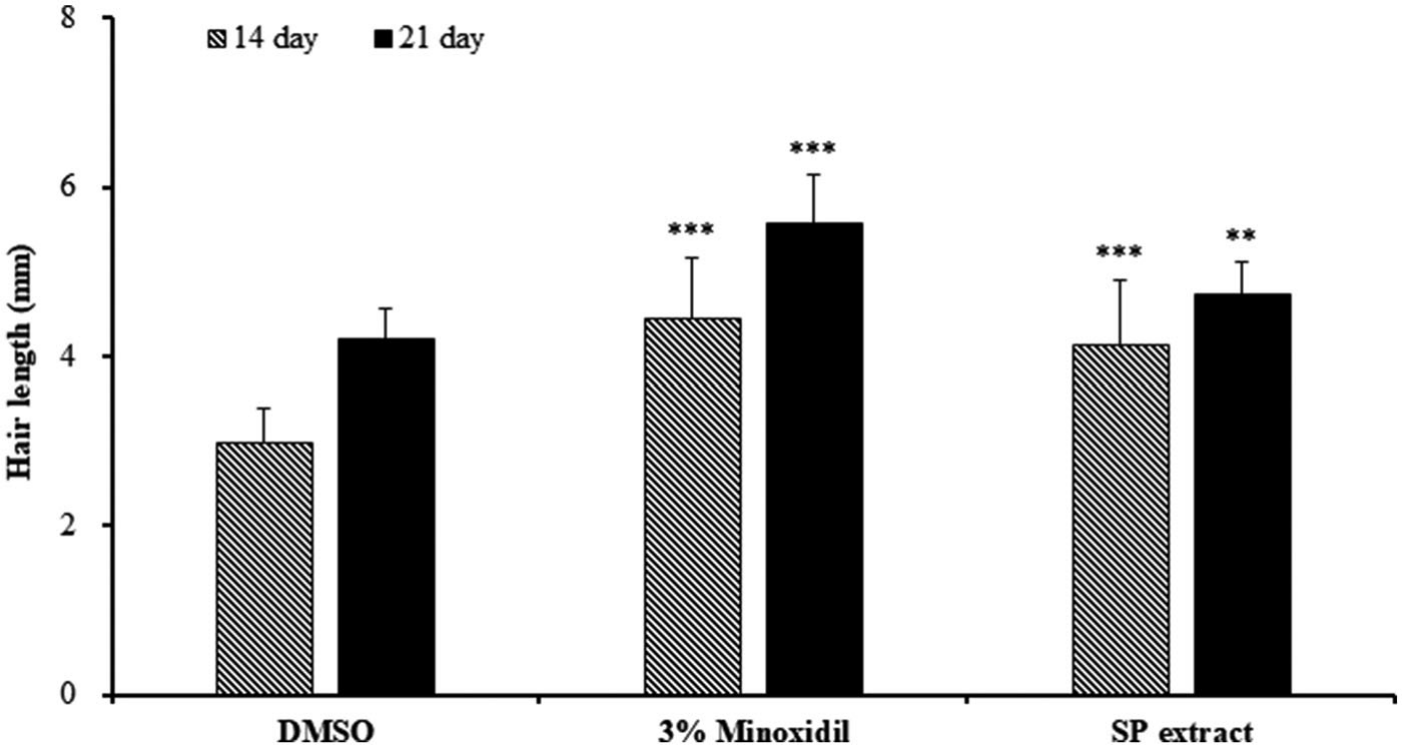
Hair length at each week after topical application of DMSO (vehicle), 3% minoxidil, and SP extract (1000 μg/mL *S. plebeia* extract dissolved in DMSO). The data shown are the mean ± SD. **p < 0.01, ***p < 0.001 compared with vehicle group. N = 6 per group.

We also randomly plucked 10 hairs from each mouse and measured the length at 14 and 21 days to confirm whether SP extract induced hair growth. At day 14, hairs of both minoxidil and SP-extract groups were significantly longer than the vehicle group (*p* < 0.001). Similarly, results were seen at 21 days (*p* < 0.001; Figure 8), indicating that the SP extract could promote hair regeneration and induce the anagen phase in the follicles of the C57BL/6 mice.

**Figure 9.**
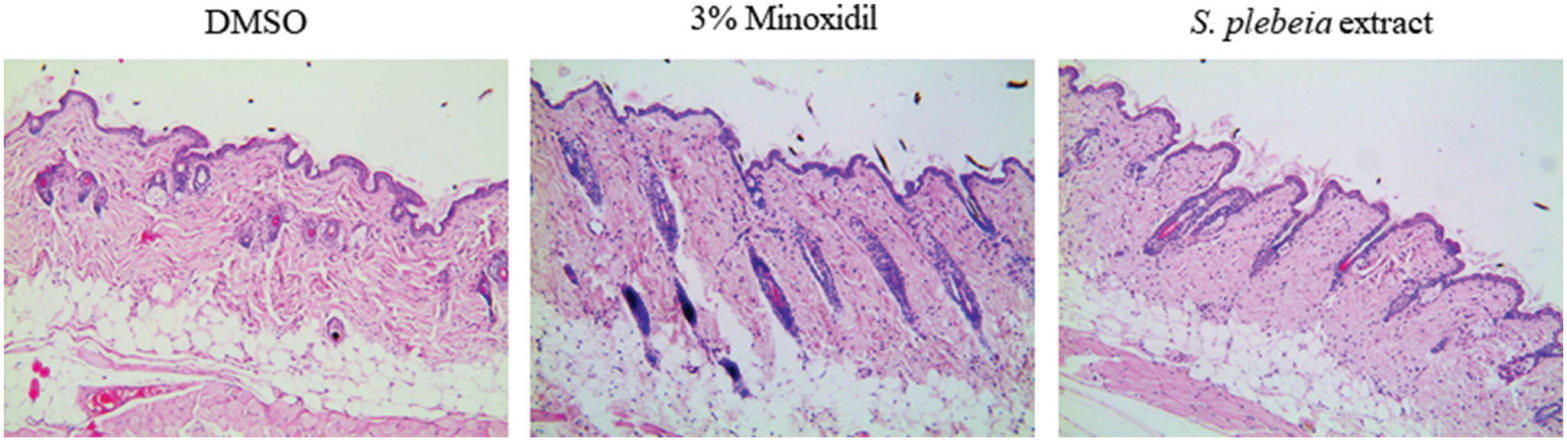
Histological change in the dorsal skin of C57BL/6 mice treated with DMSO (vehicle), 3% minoxidil, and *S. plebeia* extract (1000 μg/mL dissolved in DMSO) for 21 days. (H&E staining, 100× magnification). N = 6 per group.

After topical treatment for 3 weeks, the representative longitudinal sections of skin tissues from mice were analysed after staining with haematoxylin and eosin (Figure 9). The hair follicles were in early- to mid-anagen phase for the minoxidil group, and in early-anagen phase for the SP extract-treated group, whereas the hair follicles of vehicle group remained in the telogen phase. These observations suggest that the topical application of SP extract might promote hair growth by facilitating the conversion of telogen to early anagen phase.

## Discussion

In this study, cytotoxicity assays demonstrated that SP extract induced significant proliferation of cultured hDPCs at concentrations ranging from 3.9 to 62.5 μg/mL compared with the non-treated group (Figure 1). Also, we investigated the effect of SP extract on hDPC migration by using a scratch assay, which is particularly suited to study the stimulation of cell migration by cell interactions with extracellular matrix and cell–cell interactions (Liang et al. 2007; Enshell-Seijffers et al. 2010). It is known that dermal papilla mediates the growth and differentiation of follicular keratinocytes, the main component of hair shaft (Enshell-Seijffers et al. 2010). The number of DPC was observed to increase during the growth phase of the hair cycle and the size of DPC is well correlated with hair growth (Elliott et al. 1999). Chi et al. (2013) also reported that when the number of DPC is below a critical threshold, hair follicles with a normal keratinocyte compartment are unable to form new hairs. The proliferation and migration effects of SP extract on hDPCs might be one of the factors that maintain and elongate the anagen phase of hair cycle.

Growth factors released from DPC participate in the morphogenesis and growth cycle of hair. HGF, a multifunctional polypeptide, is secreted by hDPCs as a paracrine factor and stimulates the growth of human keratinocytes derived from the hair bulb (Shimaoka et al. 1994; Lee et al. 2001). Moreover, HGF is involved in the regulation of pigmentation, which only takes place in anagen phase of hair cycle (Slominski et al. 2004). In contrast, TGF-β1, related with catagen regulation, has been proposed to inhibit keratinocyte proliferation and induce apoptosis of hair follicle (Foitzik et al. 2000). Recent studies suggest that cultured DPC from balding scalps secrete TGF-β1/2, which suppresses epithelial cell growth in response to androgens (Inui et al. 2002). Inui et al. (2003) examined the concentration of TGF-β1 after transfecting androgen receptor expression vector into the DPC, and demonstrated that androgen-inducible TGF-β1 derived from DPC is related to AGA. TGF-β1 signalling pathway has been reported to exert biological functions via activation of the Smad2/3 cascades (Derynck and Zhang 2003). It is known to play a key role in the regulation of hair growth cycle. From our experimental results, TGF-β1 mRNA expression was considerably attenuated as well as Smad2/3 expression. Thus, we suggest that SP extract prevented anagen-catagen transition by downregulating TGF-β1 mRNA expression through suppression of the Smad2/3 cascades. The fact that in our study HGF mRNA expression significantly increased, TGF-β1 mRNA expression significantly decreased and Smad2/3 protein expression significantly attenuated in hDPCs after treatment with SP extract is favourable towards hair growth in line with the above reports (Figures 3 and 4).

Wnt/β-catenin signalling has important roles during follicular growth and is related to cell proliferation of DPCs. Ablation of β-catenin prevents hair follicle formation during epidermal embryo development, however overexpression of activated β-catenin in the epidermis results in expansion of hair follicles (Zhang et al. 2008). Recently, it was demonstrated that β-catenin-mediated transcriptional activity plays a key role in DPC function using gene expression analysis (Enshell-Seijffers et al. 2010). In addition, the clipped region in the mutant mice (conditional knockout allele of β-catenin in DP) remained largely devoid of hairs, even weeks after the hair coat had completed growth in the controls. These reports, taken together with the observation of Kishimoto et al. (2000) suggest that specific Wnt signalling pathways are sufficient to maintain the anagen state of DPCs and that the anagen phase of hair follicles can be prolonged by activating Wnt/β-catenin signalling in DPC. Other studies also reported that decreased β-catenin is observed in androgen-treated DPC among AGA patients and results in the loss of hair pigmentation (Rabbani et al. 2011; Leirós et al. 2012).

It is well known that in the absence of Wnt signalling, a destruction complex composed of adenomatous polyposis coli (APC), glycogen synthase kinase-3 (GSK3), Axin and protein phosphatase 2 A (PP2A) recruit newly synthesized β-catenin in the cytoplasm. When Frizzled and LRP receptors bind with Wnt ligands, β-catenin destruction is prevented by the inactivation of this complex, and thereby enters the nucleus and activates gene expression programmes after the stabilization and accumulation of intracellular β-catenin (Teo and Kahn 2010). We found that SP extract significantly increased the expression level of β-catenin and phospho-GSK3β, an inactivated form of GSK3β, indicating that inhibition of GSK3β activity and upregulation of β-catenin expression level are possible mechanisms by which SP extract may facilitate hair growth. Additionally, phosphorylation of ERK and Akt was significantly increased by treatment with SP extract. The role of phosphorylated ERK and Akt in mediating cell survival and apoptosis is well established (Han et al. 2004). Bcl-2 family consists of anti-apoptotic proteins such as Bcl-2 as well as pro-apoptotic proteins such as Bax (Adams and Cory 1998). Bcl-2 expressed in the dermal papilla is considered to protect hDPCs from apoptosis, while reduction in Bax protein induces hDPC growth (Adams and Cory 1998). In our study, treatment with 7.8 μg/mL SP extract caused an increase in the ratio of Bcl-2/Bax by 1.6-fold compared with NC group. Together with increased Bcl-2/Bax ratio as well as the activation of ERK and Akt pathways, it is likely to contribute to the proliferation of hDPCs when the hDPCs are treated with lower doses of the SP extract. Phosphorylated ERK and Akt are known to translocate into the nucleus, and regulate cell survival by modulating the expression of Bcl-2 and Bax (Chang et al. 2003). In addition, the main active target cell of minoxidil is the dermal papilla cells, and the probable mechanisms of action are the activation of both ERK and Akt, as well as the increase of Bcl-2/Bax ratio (Han et al. 2004). Kwon et al. (2007) also reported similar results that minoxidil plus all-trans retinoic acid promoted hair growth by facilitating the phosphorylation of ERK and Akt and increasing the ratio of Bcl-2/Bax. In light of these studies it appears that the mechanisms by which the SP extract stimulates hair growth might be similar to those of minoxidil.

C57BL/6 mouse is a widely used model for screening hair growth-promoting agents because its genetic characteristics predisposes it to longer telogen phase compared to other animal models (Shin et al. 2014), making this an appropriate model to observe the effect of SP extract on transition from telogen to anagen follicle. In our study, we observed hair growth stimulation with SP extract treatment as well as histological evidence that there was some degree of transition from telogen follicle to anagen follicle. This suggests that the transition of the hair cycle was responsible for hair growth-promoting activity of SP. In support of this is that dorsal skin tissue sections in SP extract-treated groups were observed to stay in early anagen, while the hair follicles of vehicle group remained in telogen phase.

Several other natural plant extracts have been reported to have similar positive effects on hair growth. For example, *Geranium sibiricum* L. (Geraniaceae) extract (Boisvert et al. 2017) and *Miscanthus sinensis* var*. purpurascens* Andersson (Gramineae) flower extract (Jeong et al. 2018) have exhibited propensity to not only induce the anagen phase but to maintain it. In addition, Zhang et al. (2013) observed that topical application of *Thuja orientalis* L. (Cupressaceae) extract in C57BL6/N mice induced an earlier anagen phase and prolonged the mature anagen phase, whereas the extract of *Polygonum multiflorum* Thunb. Polygonaceae has been demonstrated to increase the number and size of hair follicles in telogenic C57BL6/N mice (Park et al. 2011).

Rosmarinic acid is a polyphenolic compound first isolated from *Rosmarinus officinalis* L. (Lamiaceae), an aromatic herb used to stimulate growth of hair in folk medicine. It is known to have several biological activities, such as antioxidative, anti-inflammatory, antibacterial, and antimutagen properties (Petersen 2003; Rathi et al. 2008). Nugroho et al. (2012) demonstrated that rosmarinic acid is the main compound in the SP extract (28.5% of the methanol extract, 4.46% of dry weight). Although it is feasible that rosmarinic acid may be the active ingredient contributing to the hair growth effects of SP extract, more research will be needed to ascertain that.

## Conclusions

Our findings suggest that the SP extract regulates hDPC function possibly through ERK and Akt signalling pathways as well as activate β-catenin in the telogen phase skin, leading us to conclude that SP extract has the potential to promote hair growth, prevent hair regression, and induce early hair anagen phase. However, further studies will be needed to identify the active components of SP extract responsible for these actions and elucidate their biological activities. In addition, whether SP extract has growth-stimulation effects on other cell types of hair follicles will also need to be determined.

## Data Availability

The data used to support the findings of this study are included within the article.
